# Antenatal combination prevention for small vulnerable newborns in lower-resource settings

**DOI:** 10.1186/s44263-024-00076-z

**Published:** 2024-07-15

**Authors:** Chibuzor M. Babalola, Aamirah Mussa, Doreen Ramogola Masire, Chelsea Morroni, Jeffrey D. Klausner

**Affiliations:** 1 Department of Population and Public Health Sciences, Keck School of Medicine of University of Southern California, 1845 N Soto, Los Angeles, CA 90033 USA; 2 Botswana Harvard Health Partnership, Gaborone, Botswana; 3 Usher Institute, University of Edinburgh, Edinburgh, UK; 4 Department of Obstetrics and Gynecology, University of Botswana, Gaborone, Botswana; 5 Centre for Reproductive Health, University of Edinburgh, Edinburgh, UK

Although significant progress has been made globally, neonatal mortality persists in lower-income countries. Small vulnerable newborns—premature or growth-restricted—drive this burden, and underlying factors are multifaceted. Urgent action is needed to expedite effective interventions, prioritizing combination prevention strategies that are relevant and scalable in resource-limited settings.

## Small vulnerable newborns

When a baby is born too early or too small, it presents a significant health challenge. In 2023, the term “small vulnerable newborns” was conceptualized to emphasize the increased vulnerability of premature, small for gestational age, or low birth weight infants [1]. These infants face elevated risks of mortality, developmental delays, and chronic diseases, impacting their well-being and that of their families and imposing an economic burden on health systems and society [1]. In 2020, small vulnerable newborns accounted for one in four live births globally, with low- and middle-income countries (LMICs) bearing the greatest burden [1]. Despite progress in increasing child survival rates, half of all child deaths in the first month still occur in LMICs, particularly in Southern Asia and sub-Saharan Africa [2]. Urgent action is needed to develop and implement effective and scalable prevention strategies to reduce the risk of small vulnerable newborn births in resource-limited settings [1, 2].

## Multifaceted causality and need for combination prevention

The pathways leading to the birth of small vulnerable newborns are complex, encompassing physiological, genetic, behavioral, and environmental stressors [1]. Maternal nutrition, inflammation, and infection are modifiable factors contributing to over half of vulnerable births in LMICs [1]. Suboptimal maternal nutrition, marked by micronutrient deficiencies, can impede fetal development [3]. While some infections such as malaria, HIV, and syphilis are routinely addressed in antenatal care, others often go undetected [1, 2].

Maternal health profoundly impacts newborn health. Pregnancy is a time of increased healthcare utilization in many LMICs, so intervening on multiple modifiable risk factors during antenatal care is an opportunity to enhance maternal and newborn health. Preventive strategies targeting multiple risk factors simultaneously are required due to the complex interplay of influences on adverse pregnancy outcomes and the brief time window for intervention. These comprehensive strategies should be evidence-based, cost-effective, scalable, and sustainable.

A recent modeling estimation demonstrated that implementing eight evidence-based strategies—such as behavioral modification, nutrition optimization, and infection or inflammation mitigation during pregnancy—could annually prevent approximately 5 million preterm births across 81 LMICs [4]. Interventions may vary by pregnancy period, necessitating a combination of approaches to address multiple risks. For instance, the International Federation of Gynecology and Obstetrics recently recommended promoting a package of five essential practices around delivery to significantly improve the survival of premature infants [5].

While the fetus is still developing, during the antepartum period, a potentially promising combination strategy to address maternal malnutrition and infection or inflammation, aiming to reduce small vulnerable newborn risk, could be integrating low-dose aspirin with multiple micronutrient supplementation (MMS)—including calcium—and considering the addition of an empirically selected antibiotic such as azithromycin.

## Antenatal micronutrients

During pregnancy, the body’s nutritional demands increase to support fetal growth. MMS is a cornerstone intervention for achieving healthier pregnancies, especially in settings with limited dietary diversity and intake [3]. Modern MMS formulations, exemplified by the United Nations International Multiple Micronutrient Antenatal Preparation (UNIMMAP), were recently included in the World Health Organization (WHO) list of essential medicines [2]. MMS provides a comprehensive blend of essential vitamins and minerals at minimal cost, including iron, folate, iodine, zinc, selenium, copper, and vitamins A, C, D, E, and B [2]. Compared to standard supplementation in pregnancy, which usually includes only iron and folate (IFAS), MMS addresses a broader range of nutritional needs and has shown beneficial effects on birth outcomes [2]. In a meta-analysis including 17 trials in 14 LMICs, antenatal MMS reduced preterm births by 11%, small for gestational age births by 8%, low birth weight by 19%, and 6-month infant mortality by 29%, compared to IFAS, with greater effects seen in women who were underweight or anemic during pregnancy [6].

Several biological mechanisms support the protective effects of the micronutrients in MMS [3, 6]. Zinc, copper, and selenium bolster antioxidant enzymes, combating oxidative stress. Vitamin C aids iron absorption and enhances antioxidant defenses, alongside vitamin E. Iron supplementation prevents anemia, a key risk factor for adverse birth outcomes. Vitamin A supports embryonic development and may reduce maternal infection risk. Zinc deficiency compromises immunity crucial for fetal development. Vitamin D aids fetal structural growth, while vitamin B12 deficiency elevates homocysteine levels, which have been linked to poor pregnancy outcomes.

Interest is also growing in calcium supplementation in pregnancy, currently recommended by the WHO at high daily oral doses (1500–2000 mg) [2]. In addition to decreasing the risk of hypertensive disorders of pregnancy, calcium supplementation shows promise for reducing preterm birth. Research is exploring lower antenatal doses to address practical challenges related to cost and adherence. A recent trial found that, among women in India who also received additional micronutrients like Vitamin D_3_ as part of routine care, 500 mg of calcium was non-inferior to 1500 mg in mitigating preterm birth risk [7].

## Low dose aspirin

Well recognized for its role in the prevention of preeclampsia, low-dose aspirin (75–162 mg) has been shown to reduce preterm birth and fetal growth restriction. In a randomized controlled trial involving over 11,000 nulliparous pregnant women across six LMICs, daily 81 mg aspirin use reduced all preterm births by 11% and very preterm births by 25% compared to placebo [8]. The focus of this landmark trial on resource-limited settings demonstrates that the benefit of low-dose aspirin in pregnancy to prevent preterm birth is applicable to women in LMICs, although the generalizability of this evidence is limited to nulliparous women with singleton pregnancies.

Further research is needed to fully elucidate the mechanisms by which low-dose aspirin may reduce the risk of preterm birth, but aspirin’s anti-inflammatory, anti-platelet, and anti-thrombotic properties are well-documented, with the potential to modulate vascular function and reduce the risk of placentation disorders [8]. Importantly, concerns about adverse effects from the use of low-dose aspirin in pregnancy, such as hemorrhage, placental abruption, or birth defects, have remained largely unsubstantiated. Evidence supports a dose-dependent effect, with greater risk reduction in preterm preeclampsia documented at higher aspirin doses, up to 150 or 162 mg [9]. Regulatory bodies in higher-income countries are considering recommending higher aspirin doses in antenatal guidelines for women at high risk of preeclampsia. For feasibility considerations in LMICs, the inclusion of aspirin in scalable and cost-effective combination prevention strategies may require balancing available dosing options, cost, efficacy, and safety data. Research is planned to fill some of the gaps in evidence regarding dose-related safety for LMICs. A large multi-country study—the PEARLS trial—in Ghana, Kenya, and South Africa will quantitatively evaluate blood loss among preeclampsia-risk eligible pregnant women taking daily 75 to 150 mg aspirin [https://www.conceptfoundation.org/concept-foundation/concept-foundation-and-partners-lead-the-largest-ever-trial-study-on-use-of-aspirin-to-prevent-pre-eclampsia/]. Overall, low dose aspirin, being low-cost, readily available with a long shelf life, and tolerable, offers a potentially promising intervention to improve birth outcomes.

## Antibiotics

Maternal infection increases the risk of having a small vulnerable newborn [1]. Notably, infection-induced intrauterine inflammation is implicated in about 40% of spontaneous preterm births, more frequently attributed to ascending genital pathogens than disseminated infection. In many LMICs, HIV and syphilis are routinely screened for in pregnancy, and malaria prophylactics are provided in malaria-endemic areas. However, many infections in pregnancy remain undetected and untreated due to limited resources for adequate screening.

Choosing an appropriate antimicrobial to address a broad spectrum of infection scenarios in pregnancy remains challenging and subject to debate. And, the threat of antimicrobial resistance (AMR) must be carefully considered with any presumptive antibiotic administration, balancing the potential beneficial effects on birth outcomes against the risk of AMR. Evidence on the impact of presumptive antimicrobials in pregnancy is heterogeneous and inconclusive; a review of studies from sub-Saharan Africa suggests some benefits. Specifically, a carefully chosen empiric antibiotic with coverage for reproductive tract infections may have a role to play in reducing preterm birth and/or fetal growth restriction risks in these settings [10].

Antenatal azithromycin and cotrimoxazole are both being investigated for their potential role in preventing adverse pregnancy outcomes. A study in Zimbabwe, the COMBI trial, is assessing the effect of daily cotrimoxazole versus placebo in 1000 pregnant women to reduce preterm birth or low birth weight [https://public-ctr.mcaz.co.zw/applications/view/220]. Azithromycin covers pathogens linked to preterm birth or low birth weight, including *Chlamydia trachomatis*, *Neisseria gonorrhoeae*, *Treponema pallidum* (Syphilis), and *Plasmodium spp*. (Malaria). However, findings from research on its impact on birth outcomes vary, potentially due to different doses, frequencies, and combinations with other antibiotics. Settings, where STIs are highly prevalent, may benefit from the strategy of presumptive azithromycin in pregnancy, as a single 1 g dose clears *Chlamydia trachomatis*, the leading bacterial STI linked to preterm birth or low birth weight [11]. Also, early administration of azithromycin within 20 weeks of gestation to treat Chlamydia in pregnancy may more effectively reduce the adverse birth outcome sequelae attributable to the infection, but further research is needed. Ultimately, evidence on effectiveness, antimicrobial stewardship, and context will guide the potential inclusion of azithromycin or other antibiotics in strategies to prevent the occurrence of small vulnerable newborns in lower-resource settings.

## Conclusion

Addressing the health burden of small vulnerable newborns in lower-resource settings requires effective, feasible, scalable, and sustainable strategies. Prioritizing combination interventions to address multifaceted modifiable risks in pregnancy has promise. Targeting factors such as poor maternal nutrition, inflammation, and infections with simple-to-deliver combination regimens in routine antenatal care could be of substantial benefit.

## Data Availability

No datasets were generated or analysed during the current study.

## Abbreviations

LMIC: Low- and Middle-Income Countries
MMS: Multiple Micronutrient Supplementation
UNIMMAP: United Nations International Multiple Micronutrient Antenatal Preparation
WHO: World Health Organization
IFAS: Iron and Folic Acid
STI: Sexually Transmitted Infection

## References

[1] Ashorn P, Ashorn U, Muthiani Y, et al. Small vulnerable newborns—big potential for impact. Lancet (British edition). 2023;401(10389):1692–706. 10.1016/S0140-6736(23)00354-9. 10.1016/S0140-6736(23)00354-9 37167991

[2] WHO. WHO recommendations on interventions to improve preterm birth outcomes. 2020:1. Accessed 2024/02/01/22:19:12. https://www.paho.org/en/documents/who-recommendations-interventions-improve-preterm-birth-outcomes. 26447264

[3] Gernand AD, Schulze KJ, Stewart CP, West KP Jr, Christian P. Micronutrient deficiencies in pregnancy worldwide: health effects and prevention. Nat Rev Endocrinol. 2016;12(5):274–89. 10.1038/nrendo.2016.37. 27032981 10.1038/nrendo.2016.37 PMC4927329

[4] Hofmeyr GJ, Black RE, Rogozińska E, Heuer A, Walker N, Ashorn P, et al. Evidence-based antenatal interventions to reduce the incidence of small vulnerable newborns and their associated poor outcomes. Lancet. 2023;401(10389):1733–44. 37167988 10.1016/S0140-6736(23)00355-0

[5] Hall M, Valencia CM, Soma-Pillay P, Luyt K, Jacobsson B, Shennan A. Effective and simple interventions to improve outcomes for preterm infants worldwide: the FIGO PremPrep-5 initiative. Int J Gynaecol Obstet. 2024;165(3):929–35. 38264849 10.1002/ijgo.15269

[6] Smith ER, Shankar AH, Wu LS, et al. Modifiers of the effect of maternal multiple micronutrient supplementation on stillbirth, birth outcomes, and infant mortality: a meta-analysis of individual patient data from 17 randomised trials in low-income and middle-income countries. Lancet Glob Health. 2017;5(11):e1090–100. 10.1016/s2214-109x(17)30371-6. 29025632 10.1016/S2214-109X(17)30371-6

[7] Dwarkanath P, Muhihi A, Sudfeld CR, et al. non-inferiority of low-dose compared to standard high-dose calcium supplementation in pregnancy: study protocol for two randomized, parallel group, non-inferiority trials in India and Tanzania. Trials. 2021;22(1):838. 10.1186/s13063-021-05811-7. 34819147 10.1186/s13063-021-05811-7 PMC8611882

[8] Hoffman MK, Goudar SS, Kodkany BS, et al. Low-dose aspirin for the prevention of preterm delivery in nulliparous women with a singleton pregnancy (ASPIRIN): a randomised, double-blind, placebo-controlled trial. Lancet. 2020;395(10220):285–93. 10.1016/s0140-6736(19)32973-3. 31982074 10.1016/S0140-6736(19)32973-3 PMC7168353

[9] Horgan R, Hage Diab Y, Waller J, Abuhamad A, Saade G. Low-dose aspirin therapy for the prevention of preeclampsia: time to reconsider our recommendations? Am J Obstet Gynecol. 2023;229(4):410–8. 10.1016/j.ajog.2023.04.031. 37120049 10.1016/j.ajog.2023.04.031

[10] Ashorn P, Vanhala H, Pakarinen O, Ashorn U, Costa A. Prevention of intrauterine growth restriction and preterm birth with presumptive antibiotic treatment of pregnant women: a literature review. Nestlé Nutr Inst Workshop Ser. 2015;81:37–50. 10.1159/000365802. 26111562 10.1159/000365802

[11] Mussa A, Wynn A, Ryan R, et al. High cure rate among pregnant women in a Chlamydia trachomatis and Neisseria gonorrhoeae testing and treatment intervention study in Gaborone, Botswana. Sex Transm Dis. 2022:10.1097/OLQ.0000000000001725. 10.1097/OLQ.0000000000001725 36630419

